# Supplementary material for: Clinical Outcomes Associated with Monotherapy and Combination Therapy of Immune Checkpoint Inhibitors as First-Line Treatment for Advanced Hepatocellular Carcinoma in Real-World Practice: A Systematic Literature Review and Meta-Analysis
Source: Cancers (Basel). 2022 Dec 30;15(1):260. doi: 10.3390/cancers15010260 (PMC9818755; doi:10.3390/cancers15010260)
Supplement: Supplementary file 1 [file cancers-15-00260-s001.zip › cancers-2029040-supplementary.pdf]

Supplementary material

Clinical outcomes associated with monotherapy and combination therapy of immune checkpoint inhibitors as first-line treatment for advanced hepatocellular carcinoma in real-world practice: a systematic literature review and meta-analysis

Table S1. Study selection criteria

Study characteristic	Eligibility criteria
Patient population	➢ Adults (age ≥ 18 years) with advanced HCC
	➢ No experience of systemic treatment
	➢ Not include metastatic carcinoma
Intervention	➢ First-line ICIs-based monotherapy or combination therapy
Comparator	➢ All interventions except for first-line ICIs-based therapy
	➢ Baseline (before/after comparison)
	➢ No comparator
Outcomes	Primary outcomes
	➢ Median progression-free survival
	➢ Median overall survival
	➢ Median time to progress
	➢ Objective response rate
	➢ Disease control rate
	➢ Treatment-related adverse events
	Secondary outcomes
	➢ Prognostic factors for survival
	➢ Change of Child-Pugh score
	➢ Health-related quality of life
Study type	➢ Real-world cohort
	➢ Real-world cross-sectional
	➢ Real-world case-control
Language	➢ English

Abbreviations: HCC, hepatocellular carcinoma; ICI, immune checkpoint inhibitor.

Table S2. Search strategies

	Search terms
**1**	(liver OR hepat*).mp. [mp=ti, ab, kw]
**2**	(neoplas* OR cancer* OR tumor* OR tumour* OR carcinoma* OR oncolog* OR malign*).mp. [mp=ti, ab, kw]
**3**	(atezolizumab OR Tecentriq OR rg7446 OR mpdl3280A).mp. OR (pembrolizumab OR MK-3475 OR lambrolizumab OR Keytruda).mp. OR (nivolumab OR Opdivo OR bms-936558 OR mdx-1106).mp. OR (camrelizumab OR SHR-1210 OR AiRuiKa OR HR-301210).mp. OR (sintilimab OR Tyvyt OR IBI308).mp. OR (PD-1 OR PD-L1 OR CTLA-4 OR immune checkpoint inhibitor).mp. [mp=ti, ab, kw]
**4**	1 AND 2 AND 3

Table S3. Quality assessment of included studies

Study	Representativeness of the Exposed Cohort	Selection of the Non-Exposed Cohort	Ascertainment of Exposure	Demonstration That Outcome of Interest Was Not Present at Start of Study	Comparability of Cohorts on the Basis of the Design or Analysis	Assessment of Outcome	Was Follow-Up Long Enough for Outcomes to Occur	Adequacy of Follow Up of Cohorts	Score
Alessio et al., 2022	1	0	1	1	0	1	1	1	6
Ando et al., 2021	1	1	1	1	2	1	1	1	9
Armstrong et al., 2020*	1	0	1	1	0	1	1	1	6
Cedillo et al. 2019*	1	0	1	1	0	1	1	1	6
Chen et al., 2018*	1	0	1	1	0	1	1	1	6
Chen et al., 2021	1	1	1	1	2	1	1	1	9

Cheng et al., 2021*	1	1	1	1	0	1	1	1	7
Cheon et al., 2022	1	0	1	1	0	1	1	1	6
Chiang et al., 2021*	1	1	1	1	2	1	1	1	9
Chon et al., 2020*	1	0	1	1	0	1	1	1	6
Chuma et al., 2021	1	1	1	1	2	1	1	1	9
Cui et al., 2020	1	0	1	1	0	1	1	1	6
Dai et al., 2021	1	1	1	1	2	1	1	1	9
Dharmapuri et al. 2019*	1	0	1	1	0	1	1	1	6
Feng et al, 2017	1	0	1	1	0	1	1	1	6
Garcia et al., 2020*	1	1	1	1	2	1	1	1	9

Gaudel et al., 2021	1	0	1	1	0	1	1	1	6
Gu et al., 2020*	1	0	1	1	0	1	1	1	6
He et al., 2021	1	1	1	1	2	1	1	1	9
Himmelsbach et al., 2022	1	0	1	1	0	1	1	1	6
Huang et al., 2021	1	0	1	1	0	1	1	1	6
Iwamoto et al., 2021	1	1	1	1	2	1	1	1	9
Ju et al., 2022	1	1	1	1	2	1	1	1	9
Lee et al., 2019*	1	0	1	1	0	1	1	0	5
Lee et al., 2020	1	0	1	1	0	1	1	1	6
Li et al., 2020*	1	1	1	1	0	1	1	1	7
Liu et al., 2021	1	1	1	1	2	1	1	1	9
Liu et al., 2021	1	0	1	1	0	1	1	1	6

Mei et al., 2021	1	1	1	1	2	1	1	1	9
Rao et al., 2019*	1	1	1	1	0	1	1	1	7
Ren et al., 2022	1	1	1	1	2	1	1	1	9
Smith et al. 2020	1	0	1	1	0	1	1	1	6
Teng et al., 2021	1	0	1	1	0	1	1	1	6
Wang et al., 2021	1	1	1	1	2	1	1	1	9
Wu et al., 2021	1	0	1	1	0	1	1	1	6
Xie et al., 2021	1	1	1	1	0	1	1	1	7
You et al., 2021*	1	0	1	1	0	1	1	1	6
Zhang et al., 2022	1	0	1	1	0	1	1	1	6

*Conference abstract.

Table S4. Characteristics of included studies

Author, year	Study type	Region	Data source	Patient population				Baseline characteristics of study population			
				HBV, %	HCV, %	NAFLD, %	Alcoholic, %	Age, years (mean or median)	Male, %	Child-Pugh, %	BCLC, %
Alessio et al., 2022[1]	Retrospective	Global	Medical records (multicenter)	18.8	37.1	11.4	19.3	69	85	76 (A)/24 (B)	2 (A)/27 (B)/71 (C)
Ando et al., 2021[2]	Retrospective	Japan	EMR (single center)	65.0		NA	NA	69	75.0	100 (A)	45.0 (B)/55.0 (C)
Armstrong et al., 2020[3]*	Retrospective	USA	Medical records (single center)	30	42	NA	NA	NA	72	NA	NA
Cedillo et al. 2019[4]*	Retrospective	USA	Medical records (single center)	NA	NA	NA	NA	56.3	77.3	NA	NA

Chen et al., 2018[5]*	Retrospective	Taiwan	Medical records (single center)	49	21	NA	NA	63	79	51 (A)/26 (B)/23 (C)	NA
Chen et al., 2021[6]	Retrospective	Mainland China	Medical records (multicenter)	PLT, 54.3 LT, 61.1	PLT, 30.0 LT, 27.8	NA	NA	PLT, 58 LT, 57	PLT, 52.9 LT, 52.8	100 (A)	PLT, 67.1 (B)/32.9 (C) LT, 62.5 (B)/37.5 (C)
Cheng et al., 2021[7]*	Retrospective	Mainland China	Medical records (single center)	NA	NA	NA	NA	NA	NA	NA	10.5 (B)/89.5 (C)
Cheon et al., 2022[8]	Retrospective	Korea	Medical records (multicenter)	76.9	5.0	NA	NA	61	83.5	100 (A)	20.7 (B)/79.3 (C)
Chiang et al.,	Retrospective	Hong Kong	EMR (single center)	SBRT-IO, 75.0	NA	NA	NA	SBRT-IO, 66.5	SBRT-IO, 87.5	SBRT-IO, 87.5 (A)	SBRT-IO, 18.8

2021[9]*				TACE, 54.2				TACE, 73	TACE, 89.6	TACE, 95.8 (A)	(A)/33.3 (B)/50.0 (C) TACE, 18.7 (A)/31.3 (B)/50.0 (C)
Chon et al., 2020[10]*	Retrospective	Korea	Medical records (multicenter)	72	NA	NA	NA	60	85	NA	NA
Chuma et al., 2021[11]	Retrospective	Japan	Medical records (multicenter)	19.1	33.0	NA	NA	73	77.7	86.2 (A)/13.8 (B)	1.1 (A)/47.9 (B)/51.1 (C)
Cui et al., 2020[12]	Retrospective	Mainland China	Medical records (single	78	4	NA	NA	56	83.6	63.6 (A)/32.7	NA

			center)							(B)/3.6 (C)	
Dai et al., 2021[13]	Retrospective	Mainland China	Medical records (single center)	75.0	NA	NA	16.3	55.2	87.5	47.5 (A)/52.5 (B)	30.0 (B)/70.0 (C)
Dharmapuri et al. 2019[14]*	Retrospective	USA	Medical records (single center)	38	54	9	7	66	84	67 (A)/33 (B)	20 (B)/80 (C)
Feng et al, 2017[15]	Retrospective	Mainland China	Medical records (single center)	NA	NA	NA	NA	54.8	NA	NA	36.4 (B)/63.6 (C)
Garcia et al., 2020[16]*	Retrospective	USA	Medical records (single center)	NA	NA	NA	NA	Patients with irAEs, 69 Patients without irAEs, 70	76.7	73.3 (A)/26.7 (B)	6.7 (B)/93.3 (C)

Gaudel et al., 2021[17]	Retrospective	USA	Medical records (single center)	NA	71.4	14.3	NA	63.5	100	64.2 (A)/35.8 (B)	NA
Gu et al., 2020[18]*	Retrospective	Mainland China	Medical records (single center)	NA	NA	NA	NA	51.7	83.3	100 (A)	100 (C)
He et al., 2021[19]	Retrospective	Mainland China	EMR (multicenter)	LeToHAIC, 87.3 LEN, 90.7	NA	NA	NA	LeToHAIC, ≤ 50 y, 56.3% > 50 y, 43.7% LEN, ≤ 50 y, 48.8% > 50 y, 51.2%	LeToHAIC, 83.1 LEN, 89.5	LeToHAIC, 100 (A) LEN, 100 (A)	LeToHAIC, 100 (C) LEN, 100 (C)
Himmelsb	Retrospective	Germany	Medical	13.6	21.2	27.3	37.9	65	81.8	53.0	1.5

ach et al., 2022[20]		and Austria	records (multicenter)							(A)/34.8 (B)/7.6 (C)	(A)/33.3 (B)/53.0 (C)/12.1 (D)
Huang et al., 2021[21]	Retrospective	Mainland China	Medical records (single center)	85.0	NA	NA	NA	54.0	91.7	96.7 (A)/3.3 (B)	3.3 (A)/20.0 (B)/76.7 (C)
Iwamoto et al., 2021[22]	Retrospective	Japan	Medical records (multicenter)	13.7	37.3	NA	NA	71	88.2	92.2 (A)/7.8 (B)	47.1 (B)/52.9 (C)
Ju et al., 2022[23]	Retrospective	Mainland China	Medical records (single center)	AC, 84.6 TACE+AC, 85.7	AC, 11.5 TACE+AC, 3.6	NA	NA	AC, 55 TACE+AC, 52	AC, 84.6 TACE+AC, 82.1	AC, 78.8 (A)/21.2 (B) TACE+AC, 76.8 (A)/23.2	AC, 9.6 (B)/90.4 (C) TACE+AC, 23.2 (B)/76.8

										(B)	(C)
Lee et al., 2019[24]*	Prospective	Taiwan	Medical records (single center)	NA	NA	NA	NA	NA	NA	NA	NA
Lee et al., 2020[25]	Retrospective	Taiwan	Medical records (single center)	65.3	22.1	NA	NA	65.5	76.8	72.6 (A)/ 24.2 (B)/3.2 (C)	21.1 (B)/78.9 (C)
Li et al., 2020[26]*	Retrospective	Mainland China	Medical records (single center)	NA	NA	NA	NA	PL, 54.8<br>LEN, 53.2	NA	PL, 72.7 (A)<br>LEN, 68.1 (A)	PL, 81.8 (C)<br>LEN, 90.9 (C)
Liu et al., 2021[27]	Retrospective	Mainland China	Medical records (single center)	Cam/SOR, 94.3<br>SOR, 80.0	NA	NA	NA	Cam/SOR, 53.0<br>SOR, 53.2	Cam/SOR, 91.4<br>SOR, 83.1	Cam/SOR, 5.7 (A)/94.3 (B)<br>SOR, 12.3	Cam/SOR, 31.4 (B)/68.6 (C)<br>SOR,

										(A)/87.7 (B)	23.1 (B)/76.9 (C)
Liu et al., 2021[28]	Retrospective	Mainland China	Medical records (single center)	68.2	18.2	NA	NA	57.7	77.3	72.7 (A)/27.3 (B)	54.5 (B)/45.5 (C)
Mei et al., 2021[29]	Retrospective	Mainland China	Medical records (single center)	HPL, 82.2 PL, 76.0	HPL, 2.2	NA	NA	HPL, 49.1 PL, 50.1	HPL, 84.4 PL, 72.0	HPL, 97.8 (A)/2.2 (B) PL, 88.0 (A)/12.0 (B)	HPL, 11.1 (B)/88.9 (C) PL, 12.0 (B)/88.0 (C)
Rao et al., 2019[30]*	Prospective	USA	Medical records (single center)	NA	43	36	NA	NA	71	36 (A)/36 (B)/21 (C)	NA
Ren et al.,	Retrospective	Mainland	Medical	D-	NA	NA	NA	D-TACE-	D-	D-TACE-	D-TACE-

2022[31]		China	records (single center)	TACE-C, 74.1 C-TACE-C, 66.7				C, 53.5 C-TACE-C, 52.7	TACE-C, 85.2 C-TACE-C, 81.5	C, 88.9 (A)/11.1 (B) C-TACE-C, 85.2 (A)/14.8 (B)	C, 33.3 (B)/66.7 (C) C-TACE-C, 33.3 (B)/66.7 (C)
Smith et al. 2020[32]	Retrospective	USA	EMR (single center)		86	NA	NA	NA	83	NA	91 (C)
Teng et al., 2021[33]	Retrospective	Taiwan	Medical records (single center)	65.6	18.9	NA	NA	61.4	75.6	84.4 (A)/15.6 (B)	17.8 (B)/82.2 (C)
Wang et al., 2021[34]	Retrospective	Mainland China	Medical records (single center)	100	NA	NA	NA	50	87.9	NA	0.5 (0)/4.4 (A)/25.8 (B)/69.2

											(C)
Wu et al., 2021[35]	Retrospective	Mainland China	EMR (multicenter)	91.9	NA	NA	NA	57	90.3	NA	9.7 (A)/33.9 (B) /56.5 (C)
Xie et al., 2021[36]	Retrospective	Mainland China	Medical records (single center)	96.7	NA	NA	NA	≤ 60 y, 66.7% > 60 y, 33.3%	85.0	90.0 (A)/ 10.0 (B)	28.3 (B)/71.7 (C)
You et al., 2021[37]*	Prospective	Mainland China	Medical records (multicenter)	79.47	NA	NA	NA	NA	NA	NA	NA
Zhang et al., 2022[38]	Retrospective	Mainland China	Medical records (single center)	100	NA	NA	NA	56	88.2	82.4 (A)/17.6 (B)	47.1 (B)/52.9 (C)
Author, year	Study drug(s), n		Comparator	Follow-up,	Outcome(s) studied						NOS score
					PFS	OS	ORR	DCR	TRAEs	Prognostic	

			months						factors	
Alessio et al., 2022[1]	Atezo/Bev, n = 202	Baseline (pre-drug)	9.0	√	√	√	√	√		6
Ando et al., 2021[2]	Atezo/Bev, n = 40	Baseline (pre-drug)	3.9			√	√	√		9
Armstrong et al., 2020[3]*	Nivo, Pembro, Atezo/Bev, durvalumab, cemiplimab, n = 76	Baseline (pre-drug)	NA	√	√	√	√	√		6
Cedillo et al. 2019[4]*	Nivo, n = 22	Baseline (pre-drug)	NA		√	√	√			6
Chen et al., 2018[5]*	PD1/SOR, n = 43	Baseline (pre-drug)	NA	√	√	√	√	√	√	6

Chen et al., 2021[6]	PLT, n = 70	LT, n = 72 Baseline (pre-drug)	27.0	√	√	√	√	√		9
Cheng et al., 2021[7]*	Sintilimab/SOR, n = 31	Baseline (pre-drug)	NA	√		√	√	√		7
Cheon et al., 2022[8]	Atezo/Bev, n = 121	Baseline (pre-drug)	5.9	√		√	√	√	√	6
Chiang et al., 2021[9]*	SBRT-IO, n = 16	TACE, n = 48 Baseline (pre-drug)	SBRT-IO, 12.7 TACE, 7.4	√	√	√	√	√	√	9
Chon et al., 2020[10]*	Nivo, n = 148	Baseline (pre-drug)	NA			√			√	6
Chuma et	Atezo/Bev, n = 94	Baseline (pre-	4.7			√	√	√		9

al., 2021[11]		drug)								
Cui et al., 2020[12]	Nivo, n = 36<br>Pembro, n = 13<br>AK105, n = 6	Baseline (pre-drug)	13	√	√	√	√	√	√	6
Dai et al., 2021[13]	Sintilimab, n = 22<br>Sintilimab/SOR, n = 23<br>Sintilimab/SOR/TACE, n = 35	Between drugs<br>Baseline (pre-drug)	NA	√	√	√	√	√	√	9
Dharmapuri et al. 2019[14]*	Nivo, n = 104	Baseline (pre-drug)	17.0	√	√					6
Feng et al, 2017[15]	Nivo, n = 11	Baseline (pre-drug)	NA			√	√	√		6
Garcia et al., 2020[16]*	Nivo, n = 30	Baseline (pre-drug)	NA	√	√	√	√	√	√	9

Gaudel et al., 2021[17]	Nivo, n = 14	Baseline (pre-drug)	NA	√	√	√		√		6
Gu et al., 2020[18]*	HAIC/apatinib/toripalimab, n = 6	Baseline (pre-drug)	7.0			√	√	√		6
He et al., 2021[19]	LeToHAIC, n = 71	LEN, n = 86 Baseline (pre-drug)	NA	√	√	√	√	√	√	9
Himmelsbach et al., 2022[20]	Atezo/Bev, n = 66	Baseline (pre-drug)	6.9	√	√	√	√	√	√	6
Huang et al., 2021[21]	PD1 (Nivo, Cam, Pembro, sintilimab, toripalimab)/LEN, n = 60	Baseline (pre-drug)	NA	√		√	√	√		6
Iwamoto et al., 2021[22]	Atezo/Bev, n = 51	Baseline (pre-drug)	2.9	√		√	√	√		9

Ju et al., 2022[23]	AC, n = 52<br>TACE+AC, n = 56	Between drugs<br>Baseline (pre-drug)	13.5		√	√	√	√	√	9
Lee et al., 2019[24]*	Nivo, n = 12	Baseline (pre-drug)	NA			√	√			5
Lee et al., 2020[25]	Nivo, n = 92<br>Pembro, n = 3	Baseline (pre-drug)	5.2		√					6
Li et al., 2020[26]*	PL, n = 22	LEN, n = 22<br>Baseline (pre-drug)	PL, 6.6<br>LEN, 5.3	√		√	√	√	√	7
Liu et al., 2021[27]	Cam/SOR, n = 35	SOR, n = 65<br>Baseline (pre-drug)	8.8	√	√	√	√	√		9
Liu et al., 2021[28]	TACE/LEN/Cam, n = 22	Baseline (pre-drug)	NA	√	√	√	√	√	√	6
Mei et al., 2021[29]	HPL (Nivo, Pembro, toripalimab, sintilimab), n	Between drugs<br>Baseline (pre-	15.1	√	√	√	√	√	√	9

	= 45<br>PL, n = 25	drug)								
Rao et al., 2019[30]*	Nivo, n = 7<br>Nivo/LRT, n = 7	Baseline (pre-drug)	NA		√			√		7
Ren et al., 2022[31]	D-TACE-C, n = 27<br>C-TACE-C, n = 27	Between drugs<br>Baseline (pre-drug)	D-TACE-C, 11.0<br>C-TACE-C, 12.0	√	√	√	√	√		9
Smith et al. 2020[32]	Nivo/RT, n = 35	Baseline (pre-drug)	12.9	√	√	√	√			6
Teng et al., 2021[33]	Nivo, n = 90	Baseline (pre-drug)	8.7	√	√	√	√			6
Wang et al.,	PD1 (Cam, sintilimab, toripalimab, tislelizumab,	Baseline (pre-drug)	NA	√	√	√	√		√	9

2021[34]	Nivo, Pembro, durvalumab, Atezo)/TKIs, n = 182								
Wu et al., 2021[35]	LEN/PD1 (sintilimab, tislelizumab, Cam, toripalimab, Pembro)/TACE, n = 62	Baseline (pre-drug)	12.2			√	√	√	6
Xie et al., 2021[36]	Sintilimab/TKIs (SOR, LEN, regorafenib, apatinib), n = 60	Baseline (pre-drug)	10.4	√		√			7
You et al., 2021[37]*	Cam/TACE, n = 151	Baseline (pre-drug)	NA	√	√	√	√	√	6
Zhang et al., 2022[38]	Cam/TACE, n = 34	Baseline (pre-drug)	10.6	√	√	√	√	√	6

Abbreviations: AC, apatinib/camrelizumab; Atezo, atezolizumab; BCLC, Barcelona Clinic Liver Cancer; Bev, bevacizumab; Cam, camrelizumab; C-TACE-C, conventional transarterial chemoembolization/camrelizumab; DCR, disease control rate; D-TACE-C, drug-eluting beads transarterial

chemoembolization/camrelizumab; EMR, electronic medical records; HAIC, hepatic artery infusion chemotherapy; HBV, hepatitis B virus; HCV, hepatitis C virus; HPL, HAIC/PD1/lenvatinib; irAE, immune-related adverse event; LEN, lenvatinib; LeToHAIC, lenvatinib/toripalimab/HAIC; LRT, locoregional therapy; LT, lenvatinib/TACE; NA, not available; NAFLD, non-alcoholic fatty liver disease; Nivo, nivolumab; NOS, Nottingham Ottawa Scale; ORR, objective response rate; OS, overall survival; PD1, anti-programmed cell death 1 antibodies; Pembro, pembrolizumab; PFS, progression-free survival; PL, PD1/lenvatinib; PLT, pembrolizumab/lenvatinib/TACE; RT, radiation therapy; SBRT-IO, stereotactic body radiation therapy/nivolumab; SOR, sorafenib; TACE, transarterial chemoembolization; TKI, tyrosine kinase inhibitor; TRAE, treatment-related adverse event.

*Conference abstract.

Table S5. Probable prognostic factors for PFS and OS

Factors	PFS			OS		
	**No. of studies**	**HR (95% CI)**	***P***	**No. of studies**	**HR (95% CI)**	***P***
Gender (female vs. male)	NA	NA	NA	1	1.934 (1.010-3.706)	0.047
Age (years) (≥ 65 vs. < 65)	NA	NA	NA	1	2.630 (1.326–5.216)	0.006
AFP (ng/ml) (≥ 400 vs. < 400)	3	2.896 (1.507-5.568)	0.001	NA	NA	NA
ECOG performance status (1/2 vs. 0)	1	NA	0.021	1	NA	0.032
Child-Pugh grade (B/C vs. A)	1	NA	0.026	4	3.709 (1.239-11.099)	0.019
ALBI grade (1 vs. 2)	1	0.69 (0.48-0.99)	0.047	NA	NA	NA
BCLC stage (high vs. low)	NA	NA	NA	1	NA	NA
Tumor number (> 1 vs. 1)	NA	NA	NA	1	3.193 (1.093-9.327)	0.034
HBV DNA (IU/mL) (> 500 vs. ≤ 500)	NA	NA	NA	1	1.681 (1.033-2.735)	0.037
VB (yes vs. no)	NA	NA	NA	1	1.875 (1.134-3.100)	0.014
Albumin (g/L) (< 35 vs. ≥ 35)	NA	NA	NA	1	1.833 (1.138-2.953)	0.013
PVTT (absent vs. present)	NA	NA	NA	2	0.49 (0.27-0.87)	0.01
Macrovascular invasion (present vs. absent)	NA	NA	NA	1	2.58 (1.01-6.58)	0.048

Metastasis (absent vs. present)	2	0.63 (0.42-0.94)	0.02	2	0.44 (0.27-0.72)	0.001
irAEs (yes vs. no)	2	0.26 (0.076-0.89)	0.028	1	0.18 (0.05-0.58)	0.002
Hyperprogressive disease (yes vs. no)	1	1.947 (1.226-3.093)	NA	1	1.839 (1.108-3.055)	NA
NLR (≥ 5 vs. < 5)	2	2.23 (1.12-4.45)	0.023	2	4.68 (1.87-11.73)	< 0.001
MELD (high vs. low)	NA	NA	NA	1	NA	0.004
Best response by RECIST 1.1 (SD/PD vs. CR/PR)	1	3.10 (1.20-7.98)	0.019	NA	NA	NA
Prior local therapy/surgery (yes vs. no)	NA	NA	NA	1	0.346 (0.122–0.978)	0.045
Treatment (PL vs. HPL)	1	2.702 (1.440-5.070)	0.002	1	3.180 (1.608-6.290)	0.001
Treatment (PL vs. LEN)	1	0.58 (0.17-1.93)	NA	NA	NA	NA
Treatment (SBRT-IO vs. TACE)	1	0.10 (0.03-0.33)	< 0.001	1	0.14 (0.04-0.46)	0.001
Treatment (LeToHAIC vs. LEN)	1	0.47 (0.32-0.68)	< 0.001	1	0.39 (0.24-0.64)	< 0.001
ICI cycles (≤ 5 vs. > 5)	NA	NA	NA	1	2.644 (1.631-4.288)	0.000

Abbreviations: AFP, alpha-fetoprotein; ALBI, albumin-bilirubin; BCLC, Barcelona Clinic Liver Cancer; CR, complete response; ECOG, Eastern Cooperative Oncology Group; HBV, hepatitis B virus; HPL, hepatic artery infusion chemotherapy/PD1/lenvatinib; ICIs, immune checkpoint inhibitors; irAE, immune-related adverse event; MELD, model for end-stage liver disease; LEN, lenvatinib; LeToHAIC, lenvatinib/toripalimab/hepatic artery infusion chemotherapy; NA, not available; NLR, neutrophil-to-lymphocyte ratio; OS, overall survival; PD, disease progression; PFS, progression-free survival; PL, PD1/lenvatinib;

PR, partial response; PVTT, portal vein tumor thrombus; SBRT-IO, SBRT-IO, stereotactic body radiation therapy/nivolumab; SD, stable disease; TACE, transarterial chemoembolization; VB, virological breakthrough.

Table S6. Pooled analyses of frequently reported TRAEs

Adverse event	Any grade (%)			Grade 3-4 (%)		
	No. of cohorts	Rate (95% CI)	I^2 (%)	No. of cohorts	Rate (95% CI)	I^2 (%)
Increased AST	7	0.412 (0.262-0.563)	91	9	0.082 (0.041-0.123)	72
Platelet count decrease	8	0.375 (0.184-0.567)	97	8	0.018 (0.003-0.034)	4
Increased ALT	8	0.362 (0.224-0.500)	90	8	0.059 (0.021-0.097)	68
Hand and foot syndrome	7	0.361 (0.231-0.491)	87	6	0.045 (0.021-0.068)	0
Albumin decrease	4	0.332 (0.024-0.640)	98	3	0.008 (0.000-0.030)	0
Increased AST or ALT	3	0.322 (0.000-0.674)	98	NA	NA	NA
Anemia	4	0.317 (0.105-0.530)	98	3	0.012 (0.000-0.031)	0
Hypertension	12	0.288 (0.168-0.407)	92	16	0.040 (0.016-0.064)	63
Fatigue	20	0.277 (0.204-0.349)	85	17	0.002 (0.000-0.009)	0
Neutrophil decrease	5	0.267 (0.100-0.433)	96	5	0.017 (0.000-0.034)	42
Appetite loss	9	0.226 (0.101-0.352)	93	9	0.005 (0.000-0.016)	0
Increased bilirubin	14	0.225 (0.135-0.314)	93	12	0.013 (0.002-0.025)	23
Weight loss	3	0.210 (0.010-0.411)	89	3	0.008 (0.000-0.029)	0
Proteinuria	10	0.198 (0.134-0.263)	90	8	0.003 (0.000-0.012)	31

Fever	13	0.182 (0.093-0.271)	88	13	0.007 (0.00-0.019)	0
Pain	6	0.167 (0.032-0.302)	91	5	0.007 (0.000-0.025)	27
Hemoglobin decrease	3	0.161 (0.000-0.452)	88	NA	NA	NA
WBC decrease	6	0.135 (0.001-0.270)	94	6	0.006 (0.000-0.022)	0
Diarrhea	15	0.131 (0.082-0.179)	75	15	0.006 (0.000-0.013)	16
Abdominal pain	9	0.130 (0.062-0.199)	81	8	0.004 (0.000-0.017)	0
Nausea/vomiting	10	0.114 (0.051-0.178)	83	10	0.004 (0.000-0.014)	35
Ascites	4	0.094 (0.032-0.155)	0	3	0.051 (0.011-0.090)	28
Rash	19	0.094 (0.073-0.116)	31	17	0.006 (0.000-0.015)	1
Pruritus	9	0.091 (0.037-0.146)	68	8	0.005 (0.000-0.015)	0
Hypothyroidism	12	0.078 (0.035-0.122)	66	9	0.001 (0.000-0.009)	0
Gastrointestinal bleeding	6	0.064 (0.038-0.090)	49	6	0.051 (0.000-0.107)	77
Dyspnea/cough	7	0.032 (0.011-0.054)	0	NA	NA	NA
Edema	5	0.023 (0.003-0.044)	0	NA	NA	NA
Hyperthyroidism	4	0.014 (0.000-0.037)	44	NA	NA	NA
Increased creatinine	4	0.013 (0.000-0.049)	0	NA	NA	NA
Hepatitis	NA	NA	NA	3	0.026 (0.002-0.049)	31

Gastrointestinal perforation	NA	NA	NA	3	0.011 (0.000-0.028)	0
Myocarditis	NA	NA	NA	3	0.007 (0.000-0.026)	0

Abbreviations: ALT, alanine aminotransferase; AST, aspartate aminotransferase; NA, not available; WBC, white blood cell.

A Median PFS **B** Median OS

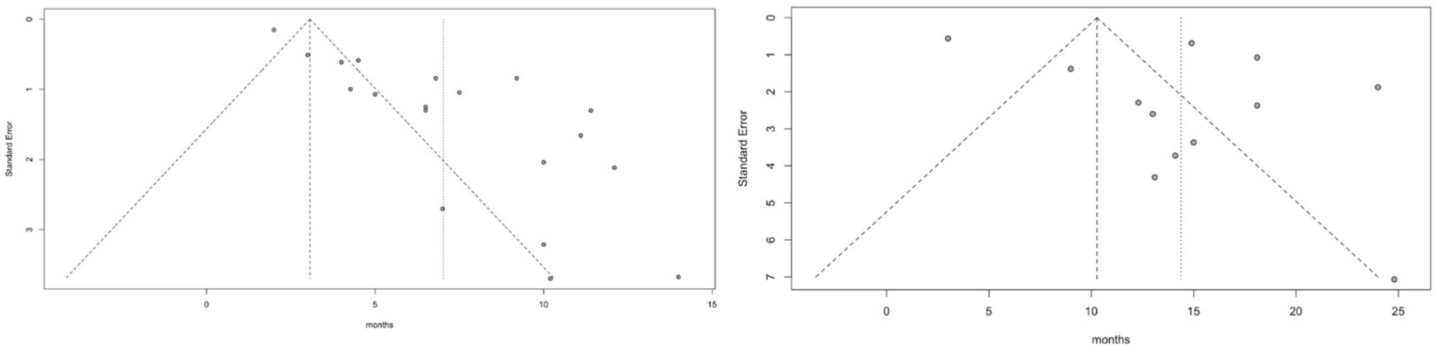

Figure S1. Funnel plots using the data of PFS and OS for assessing publication bias.

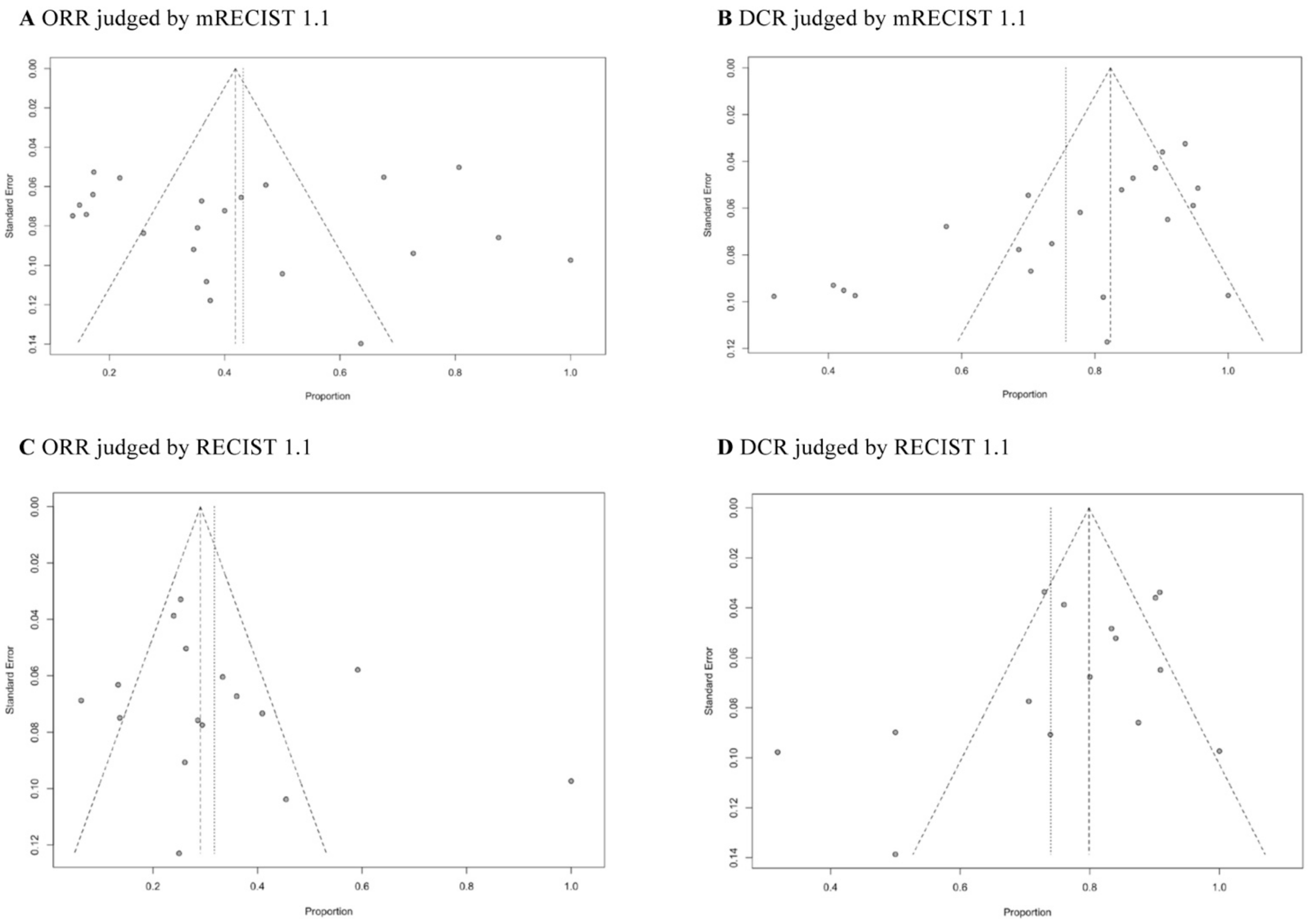

Figure S2. Funnel plots using the data of best response for assessing publication bias.

Table S7. Publication bias in the pooled analyses of PFS and OS (Egger's test)

Outcome	Egger's test
Median PFS	< 0.0001
Median OS	0.1378

Abbreviations: OS, overall survival; PFS, progression-free survival.

Table S8. Publication bias in the pooled analyses of best response (Egger's test)

Outcome	mRECIST 1.1	RECIST 1.1
ORR	0.7091	0.3252
DCR	0.0011	0.0525

Abbreviations: DCR, disease control rate; ORR, objective response rate.

Table S9. Corrected results of trim and filling method

Outcome	Publication bias	Corrected results
Median PFS	Yes	3.3041 [0.9323; 5.6758]
Median OS	No	/
ORR judged by mRECIST 1.1	No	/
DCR judged by mRECIST 1.1	Yes	0.8812 [0.7799; 0.9825]
ORR judged by RECIST 1.1	No	/
DCR judged by RECIST 1.1	No	/

Abbreviations: DCR, disease control rate; ORR, objective response rate; OS, overall survival; PFS, progression-free survival.

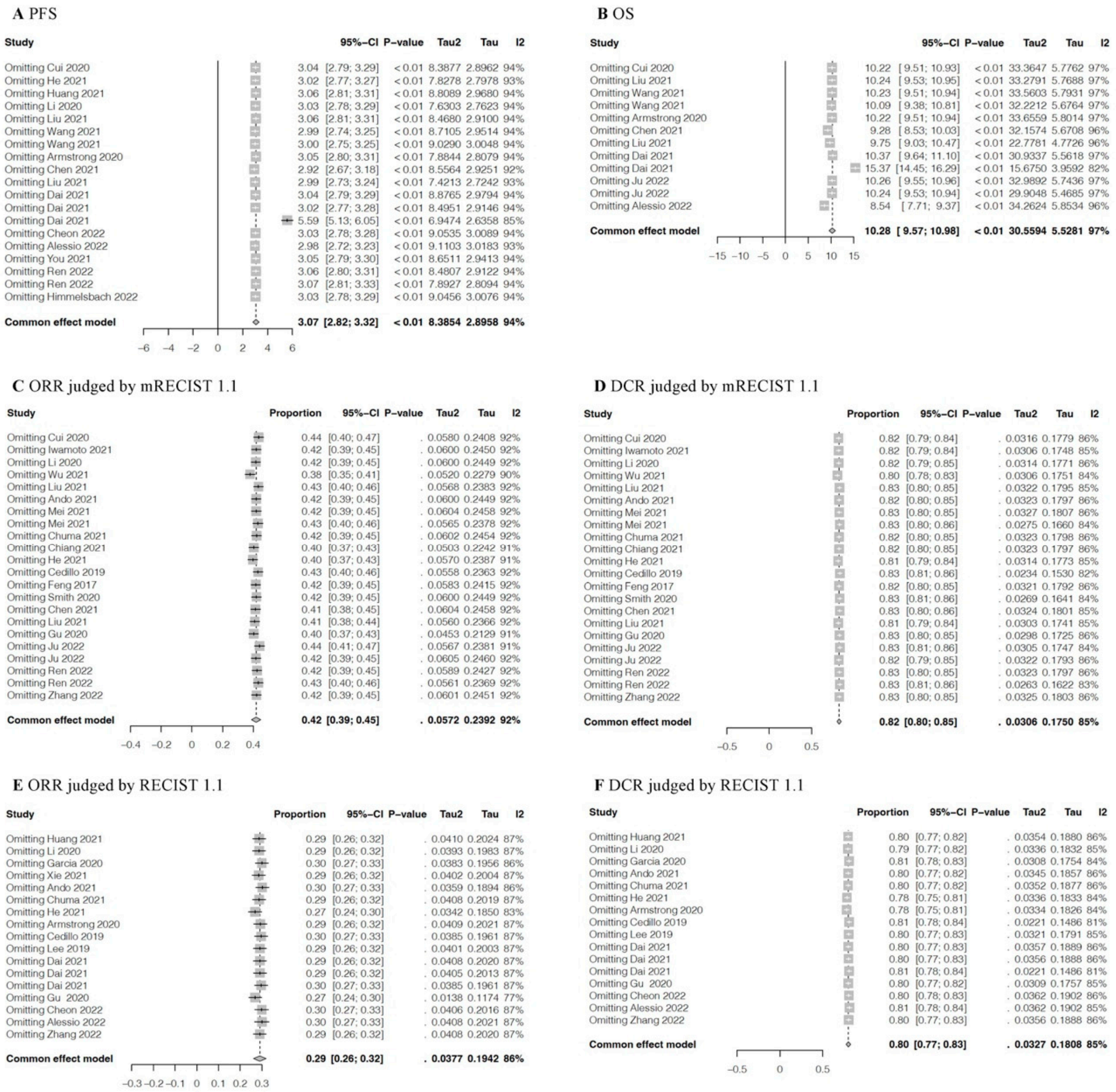

Figure S3. Sensitivity analysis.

Table S10. Meta-regression analysis for sources of heterogeneity in the estimates

Outcome	Publication year	Multicenter/single center	Viral etiology	Age	Male	Child–Pugh A	BCLC C	Sample size	Comparator	Follow-up	Quality score
Median PFS	0.0744	0.795	0.7565	0.6276	0.0988	0.2706	0.7893	0.9908	0.387	0.7099	0.068
Median OS	0.6784	0.5692	0.7392	0.8606	0.564	0.1861	0.1829	0.505	0.2216	**0.0108**	0.1046
ORR judged by mRECIST 1.1	0.3507	0.2513	0.7837	0.5453	0.2651	0.1223	0.5963	0.7008	0.3016	0.8235	0.1605
DCR judged by mRECIST 1.1	0.9159	0.1061	0.8862	0.1725	0.4589	0.7075	0.9926	0.5989	0.0675	0.0977	0.7091
ORR judged by RECIST 1.1	0.8854	0.5703	0.2792	0.0728	0.1427	0.0874	**0.0466**	0.8959	0.8729	0.6568	0.3675
DCR judged by RECIST 1.1	0.3185	0.3383	0.9734	0.7512	0.8297	**0.009**	0.8497	0.4711	0.472	0.1166	0.8121

Abbreviations: BCLC, Barcelona Clinic Liver Cancer; DCR, disease control rate; ORR, objective response rate; OS, overall survival; PFS, progression-free survival.

The table showed the meta-regression *P* value for potential sources of heterogeneity.

Table S11. Comparisons of study designs, patient characteristics, and primary outcomes between RCTs and real-world studies

	**Nivolumab**								
	**RCTs**	**Real-world studies**							
	Yau et al., 2019[39]* CheckMate 459	Cedillo et al. 2019[4]*	Chon et al., 2020[10]*	Dharmapuri et al., 2019[14]	Feng et al, 2017[15]	Garcia et al., 2020[16]*	Gaudel et al., 2021[17]	Lee et al., 2019[24]	Rao et al., 2019[30]*
Study design	Phase 3, RCT	Retrospective cohort study	Retrospective cohort study	Retrospective cohort study	Retrospective cohort study	Retrospective cohort study	Retrospective cohort study	Prospective cohort study	Prospective cohort study
Region	Global, multicenter	USA, single center	Korea, multicenter	USA, single center	Mainland China, single center	USA, single center	USA, single center	Taiwan, single center	USA, single center
Comparator	Sorafenib	NA	NA	NA	NA	NA	NA	NA	NA
N	371	22	148	67	11	30	14	12	7
Patient population	NA	NA	72% HBV	38% HBV, 54% HCV,	NA	NA	71.4% HCV, 14.3% NAFLD	NA	43% HCV, 36% NAFLD

				9% NAFLD, 7% Alcoholic					
Age, years (mean or median)	64.2	56.3	60	66	54.8	NA	63.5	NA	NA
Male, %	85	77.3	85	84	NA	76.7	100	NA	71
Child-Pugh, %	NA	NA	NA	67 (A)/33 (B)	NA	73.3 (A)/26.7 (B)	64.2 (A)/35.8 (B)	NA	0 (A)/43 (B)/43 (C)
BCLC, %	NA	NA	NA	20 (B)/80 (C)	36.4 (B)/63.6 (C)	6.7 (B)/93.3 (C)	NA	NA	NA
Median PFS (95% CI), months	3.7 (3.1-3.9)	NA	NA	16 (NA)	NA	6.2 (NA)	4 (NA)	NA	NA
Median OS (95% CI), months	16.4 (13.9-18.4)	11.33 (NA)	NA	23 (NA)	NA	12.9 (NA)	8 (NA)	NA	5 (NA)
ORR, %	15	13.6	17.6	NA	63.6	13	14.3	25	NA

DCR, %	NA	31.8	NA	NA	81.8	50	NA	50	NA
Rate of grade 3-4 TRAEs, %	22	NA	NA	NA	NA	13.3	14.3	NA	0

	Atezolizumab + Bevacizumab						
	**RCTs**	**Real-world studies**					
	Finn et al., 2020[40] IMbrave150	Alessio et al., 2022[1]	Ando et al., 2021[2]	Cheon et al., 2022[8]	Chuma et al., 2021[11]	Himmelsbach et al., 2022[20]	Iwamoto et al., 2021[22]
Study design	Open-label, Phase 3, RCT	Retrospective cohort study	Retrospective cohort study	Retrospective cohort study	Retrospective cohort study	Retrospective cohort study	Retrospective cohort study
Region	Global, multicenter	Global, multicenter	Japan, single center	Korea, multicenter	Japan, multicenter	Germany and Austria, multicenter	Japan, multicenter
Comparator	Sorafenib	NA	NA	NA	NA	NA	NA
N	336	202	16	121	50	66	19
Patient population	49% HBV, 21% HCV	18.8% HBV, 37.1% HCV,	68.8% HBV or HCV	76.9% HBV, 5.0% HCV	22.0% HBV, 28.0% HCV	13.6% HBV, 21.2% HCV,	10.5% HBV, 42.1% HCV

		11.4% NAFLD, 19.3% Alcoholic				27.3% NAFLD, 37.9% Alcoholic	
Age, years (mean or median)	64	69	69	61	72	65	71
Male, %	82	85	68.8	83.5	82.0	81.8	89.5
Child-Pugh, %	72 (A5)/28 (A6)	76 (A)/24 (B)	100 (A)	100 (A)	92.0 (A)/8.0 (B)	53.0 (A)/34.8 (B)/7.6 (C)	89.5 (A)/10.5 (B)
BCLC, %	2 (A)/15 (B)/82 (C)	2 (A)/27 (B)/71 (C)	56.3 (B)/43.8 (C)	20.7 (B)/79.3 (C)	2.0 (A)/48.0 (B)/50.0 (C)	1.5 (A)/33.3 (B)/53.0 (C)/12.1 (D)	52.6 (B)/47.4 (C)
Median PFS (95% CI), months	6.8 (5.7-8.3)	6.8 (5.2–8.5)	NA	6.5 (4.1-9.0)	NA	6.5 (4.0-9.1)	5.4 (NA)

Median OS (95% CI), months	19.2 (17.0-23.7)	14.9 (13.6–16.3)	NA	NA	NA	NA	NA
ORR, %	27.3	25	37.5	24.0	36.0	29.0	36.8
DCR, %	73.6	73	81.3	76.0	84.0	62.0	94.5
Rate of grade 3-4 TRAEs, %	56.5	28	NA	28.9	NA	59	26.3

Abbreviations: BCLC, Barcelona Clinic Liver Cancer; DCR, disease control rate; HBV, hepatitis B virus; HCV, hepatitis C virus; NA, not available; NAFLD, non-alcoholic fatty liver disease; ORR, objective response rate; OS, overall survival; PFS, progression-free survival; RCT, randomized controlled trial; TRAE, treatment-related adverse event.

*Conference abstract.

References

1. D'Alessio, A.; Fulgenzi, C.A.M.; Nishida, N.; Schonlein, M.; von Felden, J.; Schulze, K.; Wege, H.; Gaillard, V.E.; Saeed, A.; Wietharn, B., et al. Preliminary evidence of safety and tolerability of atezolizumab plus bevacizumab in patients with hepatocellular carcinoma and Child-Pugh A and B cirrhosis: A real-world study. *Hepatology* **2022**, 10.1002/hep.32468, doi:10.1002/hep.32468.
2. Ando, Y.; Kawaoka, T.; Kosaka, M.; Shirane, Y.; Johira, Y.; Miura, R.; Murakami, S.; Yano, S.; Amioka, K.; Naruto, K., et al. Early Tumor Response and Safety of Atezolizumab Plus Bevacizumab for Patients with Unresectable Hepatocellular Carcinoma in Real-World Practice. *Cancers (Basel)* **2021**, *13*, doi:10.3390/cancers13163958.
3. Armstrong, S.A.; Singh, B.P.; Kulasekaran, M.; Prins, P.; He, A.R. Predictors of immunotherapy (IO) response in hepatocellular carcinoma (HCC). *Journal of Clinical Oncology* **2020**, *38*, 555-555, doi:10.1200/JCO.2020.38.4_suppl.555.
4. Scientific Paper Abstracts Presented at the Society of Abdominal Radiology 2019 Annual Scientific Meeting and Educational Course (March 17-22, 2019, Orlando, Florida). *Abdom Radiol (NY)* **2019**, 10.1007/s00261-019-02157-2, 3210-3234, doi:10.1007/s00261-019-02157-2.
5. Chen, S.C.; Yang, M.H.; Chao, Y. Combination of Sorafenib and anti-PD-1 for advanced hepatocellular carcinoma-real world experience. *Journal for ImmunoTherapy of Cancer* **2018**, *6*, doi:10.1186/s40425-018-0422-y.
6. Chen, S.; Wu, Z.; Shi, F.; Mai, Q.; Wang, L.; Wang, F.; Zhuang, W.; Chen, X.; Chen, H.; Xu, B., et al. Lenvatinib plus TACE with or without pembrolizumab for the treatment of initially unresectable hepatocellular carcinoma harbouring PD-L1 expression: a retrospective study. *J Cancer Res Clin Oncol* **2021**, 10.1007/s00432-021-03767-4, doi:10.1007/s00432-021-03767-4.
7. Cheng, J.; Li, Y.; Wu, T.; Zhang, Z.; Chen, Y.; Su, G.; Peng, C.; Zhang, L.; Lu, Y.; Zeng, Z. Upfront or add-on combination therapeutic strategy exploration in unresectable hepatocellular carcinoma using sorafenib plus sintilimab: A retrospective analysis of real-world evidence. *Journal of Clinical Oncology* **2021**, *39*, doi:10.1200/JCO.2021.39.15_suppl.e16135.
8. Cheon, J.; Yoo, C.; Hong, J.Y.; Kim, H.S.; Lee, D.W.; Lee, M.A.; Kim, J.W.; Kim, I.; Oh, S.B.; Hwang, J.E., et al. Efficacy and safety of atezolizumab plus bevacizumab in Korean patients with advanced hepatocellular carcinoma. *Liver Int* **2022**, *42*, 674-681, doi:10.1111/liv.15102.
9. Chiang, C.L.; Chan, A.C.Y.; Chiu, W.H.K.; Kong, F.M. Combined Stereotactic Body Radiotherapy and Immunotherapy (SBRT-IO) vs. TACE in Locally Advanced Hepatocellular Carcinoma (HCC): Propensity Score Matching Analysis. *International Journal of Radiation Oncology, Biology, Physics* **2021**, *111*, e35-e36, doi:10.1016/j.ijrobp.2021.07.352.
10. Chon, H.; Kim, C.G.; Yoon, S.; Kim, C.; Kang, B.; Choi, H.J.; Lim, H.Y. Hyperprogressive disease during PD-1 blockade in patients with advanced hepatocellular carcinoma. *Journal of Clinical Oncology* **2020**, *38*, doi:10.1200/JCO.2020.38.4_suppl.550.
11. Chuma, M.; Uojima, H.; Hattori, N.; Arase, Y.; Fukushima, T.; Hirose, S.; Kobayashi, S.; Ueno, M.; Tezuka, S.; Iwasaki, S., et al. Safety and ef fi cacy of atezolizumab plus bevacizumab in patients with unresectable hepatocellular carcinoma in early clinical practice: a multicenter analysis. *Hepatol Res* **2021**, 10.1111/hepr.13732, doi:10.1111/hepr.13732.
12. Cui, H.Z.; Dai, G.H.; Guan, J.Z. Programmed Cell Death Protein-1 (PD-1)-Targeted Immunotherapy for Advanced Hepatocellular Carcinoma in Real World. *Oncotargets and Therapy* **2020**, *13*, 143-149, doi:10.2147/ott.S234868.
13. Dai, L.; Cai, X.; Mugaanyi, J.; Liu, Y.; Mao, S.; Lu, C.; Lu, C. Therapeutic effectiveness and safety

of sintilimab-dominated triple therapy in unresectable hepatocellular carcinoma. *Sci Rep* **2021**, *11*, 19711, doi:10.1038/s41598-021-98937-2.

14. Dharmapuri, S.; Özbek, U.; Lin, J.Y.; Schwartz, M.; Branch, A.; Ang, C. 759P - Outcomes of hepatocellular carcinoma (HCC) patients treated with nivolumab: The Mount Sinai Hospital experience. *Annals of Oncology* **2019**, *30*, v294, doi:https://doi.org/10.1093/annonc/mdz247.085.
15. Feng, D.; Hui, X.; Shi-Chun, L.; Yan-Hua, B.; Li, C.; Xiao-Hui, L.; Jie-Yu, Y. Initial experience of anti-PD1 therapy with nivolumab in advanced hepatocellular carcinoma. *Oncotarget* **2017**, *8*, 96649-96655, doi:10.18632/oncotarget.20029.
16. Ostios-Garcia, L.; Ramiro-Cortijo, D.; Peters, M.L.B.; Bullock, A.J. Association of immune related adverse events with superior outcomes in patients with hepatocellular carcinoma (HCC) treated with nivolumab. *Journal of Clinical Oncology* **2020**, *38*, 2.
17. Gaudel, P.; Mohyuddin, G.R.; Fields-Meehan, J. Nivolumab Use for First-Line Management of Hepatocellular Carcinoma: Results of a Real-World Cohort of Patients. *Fed Pract* **2021**, *38*, 89-91, doi:10.12788/fp.0061.
18. Gu, Y.-K.; Zhang, T.-Q.; Huang, Z.-L.; Geng, Z.-J.; Chen, C.; Li, F.-G.; Xu, L.; Sun, J.; Li, J.; Huang, Z.-M., et al. Hepatic artery infusion chemotherapy combined with apatinib and toripalimab in advanced hepatocellular carcinoma: Real-world data from a single center. *Journal of Clinical Oncology* **2020**, *38*, e16602-e16602, doi:10.1200/JCO.2020.38.15_suppl.e16602.
19. He, M.K.; Liang, R.B.; Zhao, Y.; Xu, Y.J.; Chen, H.W.; Zhou, Y.M.; Lai, Z.C.; Xu, L.; Wei, W.; Zhang, Y.J., et al. Lenvatinib, toripalimab, plus hepatic arterial infusion chemotherapy versus lenvatinib alone for advanced hepatocellular carcinoma. *Ther Adv Med Oncol* **2021**, *13*, 17588359211002720, doi:10.1177/17588359211002720.
20. Himmelsbach, V.; Pinter, M.; Scheiner, B.; Venerito, M.; Sinner, F.; Zimpel, C.; Marquardt, J.U.; Trojan, J.; Waidmann, O.; Finkelmeier, F. Efficacy and Safety of Atezolizumab and Bevacizumab in the Real-World Treatment of Advanced Hepatocellular Carcinoma: Experience from Four Tertiary Centers. *Cancers (Basel)* **2022**, *14*, doi:10.3390/cancers14071722.
21. Huang, C.; Zhu, X.D.; Shen, Y.H.; Wu, D.; Ji, Y.; Ge, N.L.; Chen, L.L.; Tan, C.J.; Zhou, J.; Fan, J., et al. Organ specific responses to first-line lenvatinib plus anti-PD-1 antibodies in patients with unresectable hepatocellular carcinoma: a retrospective analysis. *Biomark. Res.* **2021**, *9*, 11, doi:10.1186/s40364-021-00274-z.
22. Iwamoto, H.; Shimose, S.; Noda, Y.; Shirono, T.; Niizeki, T.; Nakano, M.; Okamura, S.; Kamachi, N.; Suzuki, H.; Sakai, M., et al. Initial Experience of Atezolizumab Plus Bevacizumab for Unresectable Hepatocellular Carcinoma in Real-World Clinical Practice. *Cancers* **2021**, *13*, 12, doi:10.3390/cancers13112786.
23. Ju, S.; Zhou, C.; Yang, C.; Wang, C.; Liu, J.; Wang, Y.; Huang, S.; Li, T.; Chen, Y.; Bai, Y., et al. Apatinib Plus Camrelizumab With/Without Chemoembolization for Hepatocellular Carcinoma: A Real-World Experience of a Single Center. *Front Oncol* **2021**, *11*, 835889, doi:10.3389/fonc.2021.835889.
24. Lee, P.C.; Lee, C.; Ho, H.J.; Hou, M.C.; Huang, Y.H. Association of gut microbiome with clinical response to nivolumab in advanced hepatocellular carcinoma: A pilot study. *Journal of Hepatology* **2019**, *70*, E89-E89, doi:10.1016/s0618-8278(19)30158-6.
25. Lee, P.C.; Chao, Y.; Chen, M.H.; Lan, K.H.; Lee, C.J.; Lee, I.C.; Chen, S.C.; Hou, M.C.; Huang, Y.H. Predictors of Response and Survival in Immune Checkpoint Inhibitor-Treated Unresectable Hepatocellular Carcinoma. *Cancers (Basel)* **2020**, *12*, doi:10.3390/cancers12010182.

26. Li, Q.; Chen, M.; Cao, M.; Yuan, G.; Hu, X.; Dai, W.; Zang, M.; Cheng, X.; Huang, J.; Hou, J., et al. Lenvatinib (LEN) plus anti-PD-1 antibodies vs LEN alone for advanced hepatocellular carcinoma (HCC): A real-world study. *Annals of Oncology* **2020**, *31*, S1310-S1310, doi:10.1016/j.annonc.2020.10.203.
27. Liu, Q.; You, N.; Li, J.; Wu, K.; Peng, X.; Wang, Z.; Wang, L.; Zhu, Y.; Zheng, L. Camrelizumab Plus Sorafenib Versus Sorafenib Monotherapy for Advanced Hepatocellular Carcinoma: A Retrospective Analysis. *Front Oncol* **2021**, *11*, 694409, doi:10.3389/fonc.2021.694409.
28. Liu, J.; Li, Z.; Zhang, W.; Lu, H.; Sun, Z.; Wang, G.; Han, X. Comprehensive Treatment of Trans-Arterial Chemoembolization Plus Lenvatinib Followed by Camrelizumab for Advanced Hepatocellular Carcinoma Patients. *Front Pharmacol* **2021**, *12*, 709060, doi:10.3389/fphar.2021.709060.
29. Mei, J.; Tang, Y.H.; Wei, W.; Shi, M.; Zheng, L.; Li, S.H.; Guo, R.P. Hepatic Arterial Infusion Chemotherapy Combined With PD-1 Inhibitors Plus Lenvatinib Versus PD-1 Inhibitors Plus Lenvatinib for Advanced Hepatocellular Carcinoma. *Frontiers in Oncology* **2021**, *11*, 8, doi:10.3389/fonc.2021.618206.
30. Rao, N.; Rizk, E.M.; Hwang, R.; Gartrell, R.D.; Makkar, J.; Oza, J.; Najjar, M.; Emond, J.; Fox, A.; Verna, E., et al. Nivolumab for hepatocellular carcinoma (HCC) in a real-world context. *Journal of Clinical Oncology* **2019**, *37*, 2, doi:10.1200/JCO.2019.37.15_suppl.e15675.
31. Ren, Y.; Guo, Y.; Chen, L.; Sun, T.; Zhang, W.; Sun, B.; Zhu, L.; Xiong, F.; Zheng, C. Efficacy of Drug-Eluting Beads Transarterial Chemoembolization Plus Camrelizumab Compared With Conventional Transarterial Chemoembolization Plus Camrelizumab for Unresectable Hepatocellular Carcinoma. *Cancer Control* **2022**, *29*, 10732748221076806, doi:10.1177/10732748221076806.
32. Smith, W.H.; Law, A.S.; Hulkower, M.; McGee, H.M.; Lehrer, E.J.; Schwartz, M.; Taouli, B.; Sung, M.; Buckstein, M. The effect of radiation therapy on the objective response and outcomes with nivolumab for hepatocellular carcinoma. *Acta Oncol* **2020**, *59*, 940-943, doi:10.1080/0284186X.2020.1769860.
33. Teng, W.; Lin, C.C.; Ho, M.M.; Lui, K.W.; Wang, S.F.; Hsu, C.W.; Lin, S.M. Alpha-fetoprotein response at different time-points is associated with efficacy of nivolumab monotherapy for unresectable hepatocellular carcinoma. *American Journal of Cancer Research* **2021**, *11*, 2319-+.
34. Wang, K.; Xia, Y.; Zhu, Y.; Yu, W.; Guo, Y.; Liu, L. Virological breakthrough after immune checkpoint inhibitor and nucleos(t)ide analog treatment in patients with hepatitis B surface antigen positive hepatocellular carcinoma: a real-world study. *Journal for Immunotherapy of Cancer* **2021**, *9*, e003195, doi:10.1136/jitc-2021-003195.
35. Wu, J.Y.; Yin, Z.Y.; Bai, Y.N.; Chen, Y.F.; Zhou, S.Q.; Wang, S.J.; Zhou, J.Y.; Li, Y.N.; Qiu, F.N.; Li, B., et al. Lenvatinib Combined with Anti-PD-1 Antibodies Plus Transcatheter Arterial Chemoembolization for Unresectable Hepatocellular Carcinoma: A Multicenter Retrospective Study. *J Hepatocell Carcinoma* **2021**, *8*, 1233-1240, doi:10.2147/JHC.S332420.
36. Xie, D.Y.; Sun, Q.M.; Wang, X.Y.; Zhou, J.; Fan, J.; Ren, Z.G.; Gao, Q. Immune checkpoint inhibitor plus tyrosine kinase inhibitor for unresectable hepatocellular carcinoma in the real world. *Annals of Translational Medicine* **2021**, *9*, 11, doi:10.21037/atm-20-7037.
37. You, R.; Yin, G.; Xu, H.; Zhu, X.; Zhang, Q.; Wang, Q.; Cao, C.; Lu, Y.; Jiang, H.; Liu, J., et al. Camrelizumab in combination with TACE for the treatment of unresectable hepatocellular carcinoma (HCC): A multicenter, single-arm, prospective real-world study. *Journal of Clinical*

Oncology **2021**, *39*, e16114-e16114, doi:10.1200/JCO.2021.39.15_suppl.e16114.

38. Zhang, J.X.; Chen, P.; Liu, S.; Zu, Q.Q.; Shi, H.B.; Zhou, C.G. Safety and Efficacy of Transarterial Chemoembolization and Immune Checkpoint Inhibition with Camrelizumab for Treatment of Unresectable Hepatocellular Carcinoma. *J Hepatocell Carcinoma* **2022**, *9*, 265-272, doi:10.2147/JHC.S358658.
39. Yau, T.; Park, J.W.; Finn, R.S.; Cheng, A.L.; Mathurin, P.; Edeline, J.; Kudo, M.; Han, K.H.; Harding, J.J.; Merle, P., et al. LBA38_PR - CheckMate 459: A randomized, multi-center phase III study of nivolumab (NIVO) vs sorafenib (SOR) as first-line (1L) treatment in patients (pts) with advanced hepatocellular carcinoma (aHCC). *Annals of Oncology* **2019**, *30*, v874-v875, doi:https://doi.org/10.1093/annonc/mdz394.029.
40. Finn, R.S.; Qin, S.; Ikeda, M.; Galle, P.R.; Ducreux, M.; Kim, T.Y.; Kudo, M.; Breder, V.; Merle, P.; Kaseb, A.O., et al. Atezolizumab plus Bevacizumab in Unresectable Hepatocellular Carcinoma. *N Engl J Med* **2020**, *382*, 1894-1905, doi:10.1056/NEJMoa1915745.
